# Effects of Geographical Origin and Timing of Broodstock Collection on Hatchery Conditioning of the Clam *Ruditapes decussatus* (L. 1758)

**DOI:** 10.3390/ani15010029

**Published:** 2024-12-26

**Authors:** Rania Azirar, Samah Fettach, Fiz da Costa, Montse Pérez, Abderrahim Chiaar, Adil Aghzar, Yassine Ouagajjou

**Affiliations:** 1 Research Team of Agriculture and Aquaculture Engineering (G2A), Polydisciplinary Faculty of Larache, Abdelmalek Essaadi University, Tetouan 93000, Morocco; rania.azirar@etu.uae.ac.ma (R.A.); a.aghzar@uae.ac.ma (A.A.); 2 Amsa Shellfish Research Station, National Institute of Fisheries Research (INRH), Tetouan 93000, Morocco; fettach@inrh.ma (S.F.); ouagajjou@inrh.ma (Y.O.); 3 AquaCOV, Centro Oceanográfico de Vigo, Instituto Español de Oceanografía (IEO, CSIC), 36390 Vigo, Spain; fiz.dacosta@ieo.csic.es; 4 Center of Pathology of Aquatic Animals, National Institute of Fisheries Research (INRH), Tangier 90000, Morocco; chiaar@inrh.ma

**Keywords:** *Ruditapes decussatus*, broodstock, geographical origin, season, gonadal development, hatchery, Morocco

## Abstract

The grooved carpet shell clam (*Ruditapes decussatus*), widely found along Morocco’s coasts and estuaries, is a key economic resource due to its high market value. However, clam production has declined over recent decades, largely due to the overexploitation of natural beds, and recruitment failures, leading to a limited wild spat availability. This study examined how the broodstock collection season (winter vs. summer) and origin (South Atlantic vs. North Mediterranean) affect broodstock performances in hatcheries. The maturity development (condition index (CI) and gonadal condition index (GCI)), histological examination, and reproductive output were evaluated. The results showed that Mediterranean clams achieved a higher maturity during winter conditioning (CI = 13.60 ± 1.02, GCI = 6.01 ± 0.90) than the Atlantic population (CI = 11.51 ± 1.50, GCI = 5.31 ± 1.14). Moreover, Mediterranean clams produced more oocytes per female (2.34 million), despite the lower spawning rate (42%), compared to the Atlantic clams (1.68 million) with a 69% spawning rate by the end of the winter conditioning. These findings highlight the importance of selecting broodstock by geographic origin to optimize shellfish hatchery production.

## Simple Summary

Optimizing production strategies for clam farming is crucial to achieve economic and biological sustainability. Improving broodstock-conditioning conditions, such as the geographical origin, timing of collection, temperature, and nutrient levels, can significantly enhance outcomes. Developing Moroccan hatcheries rearing protocols adapted to local environmental factors promotes sustainable aquaculture, making clam farming a more feasible and profitable industry for the country. Effective broodstock conditioning is critical for producing high-quality larvae and spat in bivalve hatcheries. This study aimed to assess the impact of the geographic origin and season of collection on broodstock-conditioning performances to design optimal clam rearing programs.

## 1. Introduction

Shellfish, including clams, are gastronomically much appreciated and sought worldwide, representing a very significant commercial value [1]. In 2017, the global shellfish market (mussels, clams, oysters, and scallops) reached €30.1 billion, with the clam sector alone accounting for €9.77 billion [2]. Furthermore, clam production faces substantial challenges due to human-induced activities such as overexploitation [3], habitat degradation [4,5], environmental pollution [6], alien species introduction [7], and climate-related factors, including water warming [8], salinity fluctuations [9], and ocean acidification [9,10]. Additionally, a critical issue arises from the absence of population resistance to infections caused by pathogenic organisms including bacteria, viruses, or protists [11], leading to extremely high mortality rates of 60–90% for juveniles in natural beds [12]. The widespread occurrence of emerging diseases like perkinsosis, caused by the protozoan parasite *Perkinsus olseni* and disadvantageous environmental conditions [13,14,15,16] has negatively affected clam species, namely, *Ruditapes decussatus* and *Ruditapes philippinarum*, provoking increased mortality rates and leading to significant economic impacts.

*Ruditapes decussatus* (L. 1758), commonly known as the “grooved carpet shell clam”, is one of the most important bivalve species widely distributed along the coastal and estuarine areas of Europe and Northern Africa [17,18]. Due to its high bio-ecological and commercial fisheries incomes, it is proposed as a promising candidate for the emerging bivalve aquaculture improvement in Mediterranean lagoons [19]. Clam spat production in hatcheries is currently the only sustainable alternative for the aquaculture activities facing the infaunal overexploitation accrued in natural populations [20,21,22]. The growing market demand and the decline of the natural stocks have generated a great interest regarding in-captivity spat production.

In Morocco, this species has been heavily overexploited for several decades along the Moroccan coastlines [23]. To address this alarming situation, artificial spawning and larval rearing programs could provide an alternative source of spat [1]. To improve rearing programs for *Ruditapes decussatus*, detailed studies on the species’ reproductive cycle and spawning periods in the wild have been carried out in many countries, such as Morocco, Tunisia, Spain, and Portugal [17,24,25,26]. In two Atlantic lagoons (Oualidia and Mulay Bousselham lagoons) in Morocco, the spawning period of *Ruditapes decussatus* typically occurs during two main periods: May–June and August–September [24]. In the populations investigated in this study, Kamara et al. [27] mentioned that in the Atlantic region (Dakhla), there are two distinct spawning periods, from March to May and from July to September, whereas, the reproductive cycle of *R. decussatus* in the Mediterranean area revealed a single gamete release period extending from May to November, peaking in July.

Although there has been continuous progress in bivalve’s hatchery knowledge, there are still several biological aspects that remain unknown or poorly understood [28], such as the effect of the geographical broodstock origin and timing of broodstock collection on the maturity development and spawning success of *Ruditapes decussatus*. Regarding the geographical location, Morocco’s climate exhibits a diverse range of characteristics. Along the northern coast, it tends to be temperate, featuring mild winters and hot, dry summers [29]. In addition, seasonal energy storage and utilization in marine bivalves are strongly linked to their reproductive activity and geographical location [26,30,31,32].

Production of *Ruditapes decussatus* spat is a pivotal sector for which most techniques have been developed through practical experimentation, modifying approaches across species and evaluating their impact on growth, reproduction, and survival [1]. Furthermore, hatchery production is still affected by several constraints, which are often species-specific and are encountered at different stages in the biological cycle of mollusks [33]. The techniques commonly used in hatcheries include three stages: (i) broodstock conditioning, (ii) larval rearing, and (iii) spat production. Although, these stages, routinely carried out in hatcheries, remain empirical, and the knowledge related to the reproductive physiology of *Ruditapes decussatus* is limited.

Broodstock conditioning is one of the most crucial aspects that is influenced by several factors, such as the temperature [17,20,34,35,36] and photoperiod [1,37,38], which directly affect metabolic rates and reproductive behavior and cycles in many aquatic species. Moreover, the food (microalgae) quality and availability are fundamental determinants in terms of reproductive success during broodstock maintenance [23]. However, the time required for broodstock conditioning to develop mature oocytes depends on the gonad maturation stage of individuals at the time of collection [39]. Those individuals collected at different stages of gonadal development may need varying amounts of time to reach full maturity. Therefore, the initial gametogenic stage of a given clam population may differ based on the localities of collection, environmental conditions [17,40,41], and season of collection [17]. These factors need to be investigated due to their importance in broodstock conditioning. Although, few authors have carried out research recently on the impact of these factors on clams, especially on *Ruditapes decussatus* [17]. To promote clam hatcheries in North Africa, particularly in Morocco, there is a noticeable lack of information. Consequently, more scientific research is needed to advance spat production for this species.

The Moroccan clam farming industry has a high demand for *R. decussatus.* Thus, in order to support local hatchery production, two geographical areas were selected based on the clam occurrence and zoo-sanitary status, one in the North (Mediterranean Sea) and one in the South (Atlantic Ocean). Precisely, the study aimed to investigate the effect of the geographic origin and timing of the broodstock collection on *R. decussatus* broodstock maturation under controlled conditions to maximize the spawning output.

## 2. Materials and Methods

### 2.1. Broodstock Collection

**Figure 1:**
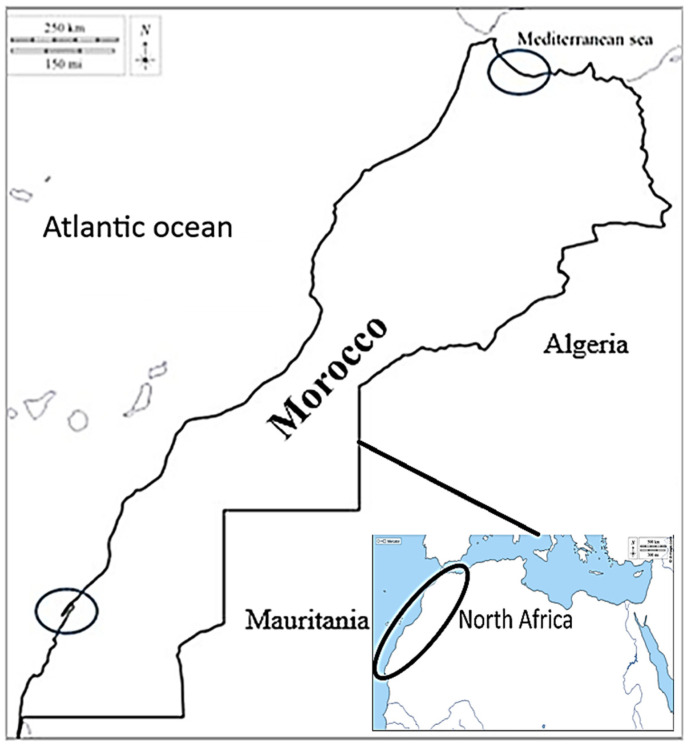
Collection places for broodstock of the grooved carpet shell clam *Ruditapes decussatus*. North, Mediterranean Sea (M’diq) and South, Atlantic Ocean (Dakhla).

The broodstock samples were hand-collected from two geographical distinct areas during two different thermal seasons (winter and summer). The specimens of *Ruditapes decussatus* were collected in 2023 from the northern area (North Mediterranean: M’diq 35°41′09″ N, 5°19′31″ W), with an average shell-length of 38.02 ± 0.40 mm and average live weight of 9.51 ± 0.22 g. Individual samples from the southern area (South Atlantic: Dakhla 23°43′ N 15°57′ W) were collected during 2022, with an average shell-length of 41.03 ± 2.84 mm and average live weight of 10.54 ± 1.27 g (Figure 1). The samples were collected from Dakhla (southern Morocco, Atlantic Ocean) during winter (January 2022, temperature = 17.0 °C, and daylight ≃ 10 h) and during summer (June 2022, temperature = 18.5 °C, and daylight ≃ 13 h). Moreover, the samples from M’diq (northern Morocco, Mediterranean Sea) were collected during winter (January 2023, temperature = 17.5 °C, and daylight ≃ 10 h) and during summer (June 2023, temperature = 18.6 °C, and daylight ≃ 14 h).

Upon arrival, the clams were counted and cleaned to remove any epifauna before being placed into 500 L tanks emerged with seawater at the same temperature of the natural beds where they were collected (see above). After five days of quarantine depuration (acclimatation), samples of 25 clams were collected before the conditioning (week zero, W0) to evaluate their initial state of maturity and gonadal development. For all clams’ groups, a total of 135 individuals were divided into pseudo-replicates (4 g/L) and placed in perforated baskets within a 500 L tank.

During the conditioning, water parameters were daily monitored to optimize the gametogenic development. The temperature of the Mediterranean and Atlantic clams was gradually increased by 0.3 °C per day until it reached 22.03 ± 0.30°C, as described by other authors [17]. The seawater was filtered at 0.2 µm, UV-disinfected, and its temperature was regulated by means of a heat exchanger. The clams were reared under a closed system (CS) under a controlled photoperiod for both localities and seasons (9 h light/15 h dark) and the water was changed every 48 h. Physicochemical parameters such as salinity (35 ppt), pH (8.03 ± 0.02), and dissolved oxygen (5.10 ± 0.40 mg/L) were monitored throughout the experiment. Daily mortality checks were conducted, and any deceased clams were promptly removed to prevent contamination and further loss.

### 2.2. Feeding Process

Two microalgae strains, *Isochrysis galbana* (CCAP 927/1) and *Chaetoceros calcitrans* (CCAP 1085/3), were used during this study based on their high nutritional value [42,43]. These microalgae were batch-cultured in 100 L polycarbonate tanks using F/2 media under continuous aeration, a temperature of 24.01 ± 1.02 °C, and salinity levels of 36 ppt and 30 ppt for *I. galbana* and *C. calcitrans,* respectively. The clams were fed twice a day with a bi-specific mixture of 25% *I. galbana* and 75% *C. calcitrans,* as mixed microalgae diets are generally considered more beneficial for bivalves [44], especially clams [22,23,45]. The daily amount of microalgae (dry weight) fed to clams was estimated based on a ratio of 3.5% of the dry meat weight of clams [46].

### 2.3. Broodstock Conditioning

**Figure 2:**
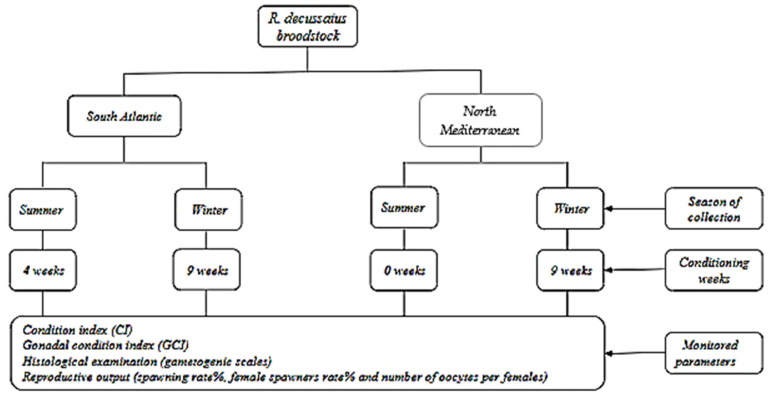
Experimental design of the grooved carpet shell clam *Ruditapes decussatus* broodstock conditioning per season and origin.

During winter, the conditioning lasted for nine weeks for both localities (Atlantic and Mediterranean populations). However, in summer, the Atlantic population was conditioned for four weeks, while the Mediterranean population required no conditioning due to the successful spawning induction occurring at week zero (W0). The sampling took place at the start (week zero) and at the end of each conditioning (week nine for winter and week four for summer). At each sampling, 25 individuals were collected to evaluate the condition index (CI) and gonadal condition index (GCI). Although, 10 individuals were collected for histological examinations (Figure 2).

### 2.4. Physiological Indices

The condition index (CI) of the clams was calculated according to Walne and Mann [47], using the following formula:

CI = [dry weight of the flesh (g)/dry weight of the shell (g)] × 100(1)

where the flesh and the shell were separated, dried in the oven at 75 °C for 24 h, and weighed to calculate their dry weight.

The gonadal condition index (GCI) was calculated according to Ojea et al. [26], using the following formula:

GCI = [dry weight of the gonadal visceral mass (g)/dry weight of the shell (g)] × 100(2)

The gonadal–visceral mass was separated using a scalpel blade, dried in the oven at 75 °C for 24 h, and weighed to calculate the dry weight.

### 2.5. Histological Examination of the Gonads

The soft tissues were fixed in Davison solution for 48 h, and then transferred to 70% ethanol for storage. Tissues from these samples were dehydrated with serial dilutions of ethanol and embedded in paraffin. Thick sections (2 μm) were cut on a microtome and stained with haematoxylin and eosin. The histologically prepared slides were examined using a microscope at a 40(×) magnification and each specimen was assigned to a stage which represented the gonadal state. The clam reproductive maturity was categorized into six stages adapting the gametogenic scale proposed by Delgado and Pérez-Camacho [48]. Stage I = resting stage, stage II = initiation of gametogenesis, stage III = advanced gametogenesis, stage IV = ripe, stage V = partially spawned, and stage VI = spent. When multiple stages occurred concurrently within a specific section, the classification of the stage was determined by the prevailing condition of the majority of follicles found in that section.

### 2.6. Spawning

The spawning induction was carried out using the thermal shock technique at the end of each conditioning period, except for the Mediterranean clams during summer, which was performed at week zero. This involved four alternating cycles of cold seawater (10–12 °C) for 30 min and warm seawater (26–28 °C) for 1 h. Once the clams began releasing gametes, each spawning individual (male or female) was placed individually in a small container of 0.5 L (0.2 µm filtered and UV-treated seawater), maintained at 22 ± 1 °C to facilitate the continued gamete release. Sperm and oocytes were then filtered through a 20 µm mesh sieve, and the number of oocytes was estimated using an optical microscope.

### 2.7. Statistical Analysis

The condition index (CI), gonadal condition index (GCI), and number of oocytes released per female were examined by a one-way analysis of variance (ANOVA) (Fisher test), to evaluate the effect of the geographical broodstock origin and timing of broodstock collection (winter and summer). Results were considered significant at *p* ≤ 0.05. The data analysis was carried out using logical R (interface Rcmdr) version R 4.2.1.

## 3. Results

### 3.1. Condition Index (CI)

**Figure 3:**
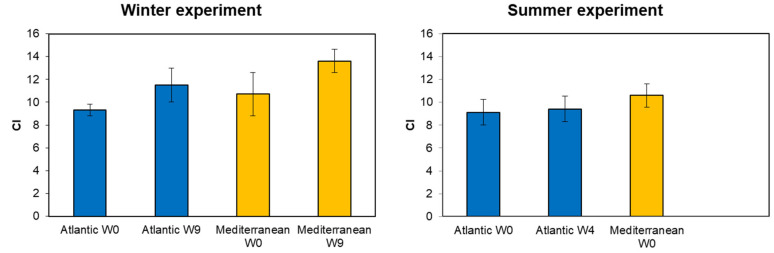
Condition index (CI) of the two grooved carpet shell clam *Ruditapes decussatus* populations (Atlantic and Mediterranean) (mean ± SD, *n* = 25) conditioned at different seasons (winter and summer). W0: week zero (start of the conditioning); W4: four weeks of conditioning; and W9: nine weeks of conditioning.

In winter, the Atlantic clam group’s CI increased from 9.30 ± 0.51 at week zero (W0) to 11.51 ± 1.50 by the end of the conditioning (week nine W9, Figure 3). Similarly, the Mediterranean population’s CI increased from 10.71 ± 1.90 at W0 to 13.60 ± 1.02 by W9. During summer, the Atlantic population showed a slight increase in CI, from 9.12 ± 1.11 at week zero to 9.42 ± 1.10 at week nine. However, for the Mediterranean population, no conditioning was conducted due to the successful spawning induction at week zero (CI = 10.60 ± 1.02). A one-way ANOVA analysis showed a highly significant effect of origin on CI at the end of the winter conditioning (week 9) (F = 15.95, *p* = 0.00018). Also observed was a highly significant effect of the season of collection on CI for the southern population (Atlantic) at the end of the conditioning (F = 16.85, *p* = 0.00023). The effect of the season of collection on CI was only analyzed in the southern population (Atlantic), since for the northern population (Mediterranean), only winter conditioning was carried out. No significant effect was found on CI between the Atlantic and Mediterranean population during winter at week 0 (F = 5.87, *p* = 0.114). Furthermore, a significant effect was revealed between both populations during summer at week 0 (F = 6.71, *p* = 0.0029).

### 3.2. Gonadal Condition Index (GCI)

**Figure 4:**
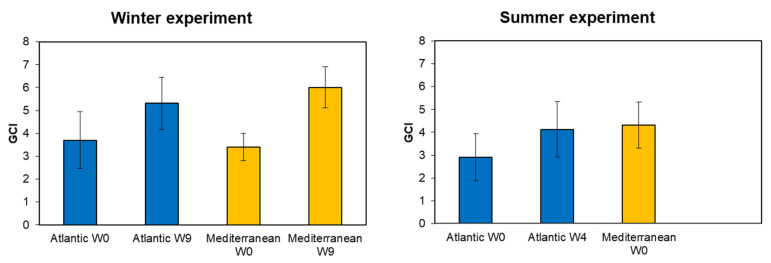
Gonadal condition index (GCI) of grooved carpet shell clam *Ruditapes decussatus* (Atlantic and Mediterranean origin) (mean ± SD, *n* = 25) conditioned at different seasons (winter and summer). W0: week zero (start of conditioning); W4: four weeks of conditioning; and W9: nine weeks of conditioning.

In winter, the gonadal condition index (GCI) increased from week zero to week nine for both populations (Figure 4). In the Atlantic population, the GCI rose from 3.70 ± 1.23 at week zero to 5.31 ± 1.14 by week nine, while the Mediterranean population showed a greater increase from 3.40 ± 0.60 to 6.01 ± 0.90 over the same period. During summer, only four weeks of conditioning were needed for the Atlantic population to reach a GCI of 4.13 ± 1.21. No conditioning was carried for the Mediterranean clams due to the successful spawning induction at week zero (GCI = 4.31 ± 1.01). A one-way ANOVA analysis showed a significant effect of the origin on the GCI at the end of the winter conditioning (Week 9) (F = 14.05, *p* = 0.00021). A significant effect of the season of conditioning was shown on the GCI for the southern population (Atlantic) at the end of the conditioning (F = 25.12, *p* = 0.00037). The effect of the season of collection on the GCI was only analyzed in the South Atlantic population, since for the North Mediterranean, only winter conditioning was carried out. No significant effect was shown between the Atlantic and Mediterranean population on the GCI during winter at week 0 (F = 5.12, *p* = 0.126). However, a highly significant effect was revealed between both populations during summer at week 0 (F = 16.07, *p* = 0.00019).

### 3.3. Gametogenic Scales

**Figure 5:**
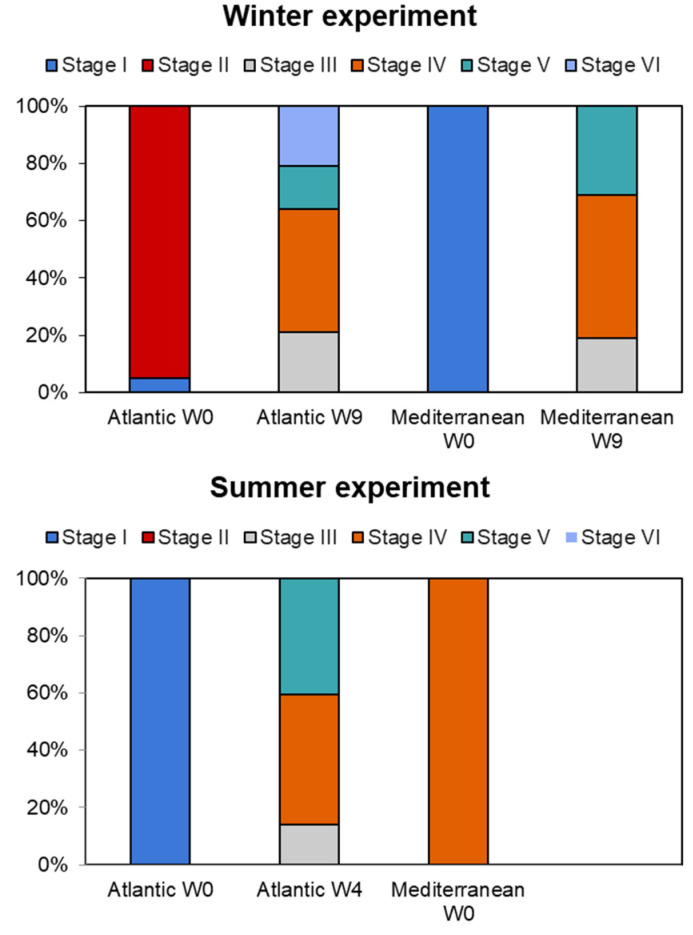
Gonadal development phases of the two grooved carpet shell clam *Ruditapes decussatus* populations (Atlantic and Mediterranean) conditioned at different seasons (winter and summer). Stage I = resting stage, stage II = initiation of gametogenesis, stage III = advanced gametogenesis, stage IV = ripe, stage V = partially spawned, and stage VI = spent.

Water temperature can significantly influence the gonadal state at the beginning of experiments, as it often serves as a key environmental cue for reproductive cycles in many species [20,34]. The gonadal state was significantly influenced by temperature and the interaction between temperature and time. At the start of the experiment (week zero), the Atlantic and Mediterranean populations showed different stages of gonadal development across the two seasons. Both populations displayed gonads at stage I (resting stage), stage II (initiation of gametogenesis), and stage IV (ripe). In summer, the Atlantic population revealed 43% of the clams at the ripe stage (stage IV), followed by 38% at stage V (partially spawned), and 19% at stage III (advanced gametogenesis) by the end of the conditioning (week nine) (Figure 5). In contrast, the Mediterranean population was dominated by individuals at stage IV (100%), accounting for the successful spawning induction at week zero. However, during the winter conditioning, a significant maturation was observed over nine weeks of conditioning. Atlantic clams exhibited a more pronounced shift in stage distribution compared to the Mediterranean clams. However, the maturation process in the Mediterranean population was faster than in the southern clams, with 50% reaching ripe stage (stage IV), compared to 43% in stage IV for Atlantic clams at the end of conditioning (Figure 5).

Clams, like many other marine organisms, exhibit seasonal patterns of activity that are tightly linked to water temperature. During cooler periods (winter season), clams often enter a resting state, slowing down metabolic processes to conserve energy which needs more time (weeks) to achieve the maturity state of gonads (nine weeks to achieve maturity for both localities during winter season). However, warmer temperatures (summer period) can trigger more active feeding related with the abundance of chlorophyl in the natural bed and reproductive behaviors, potentially disrupting the resting state, which explains the short period of conditioning needed for the Atlantic population (four weeks). Furthermore, Mediterranean clams showed a successful spawning at the start of the summer experiment due to the initial gametogenic state (stage IV: ripe) occurring due to the environmental conditions of the area.

#### Sexual Differentiation and Aspects of Gametogenesis

**Figure 6:**
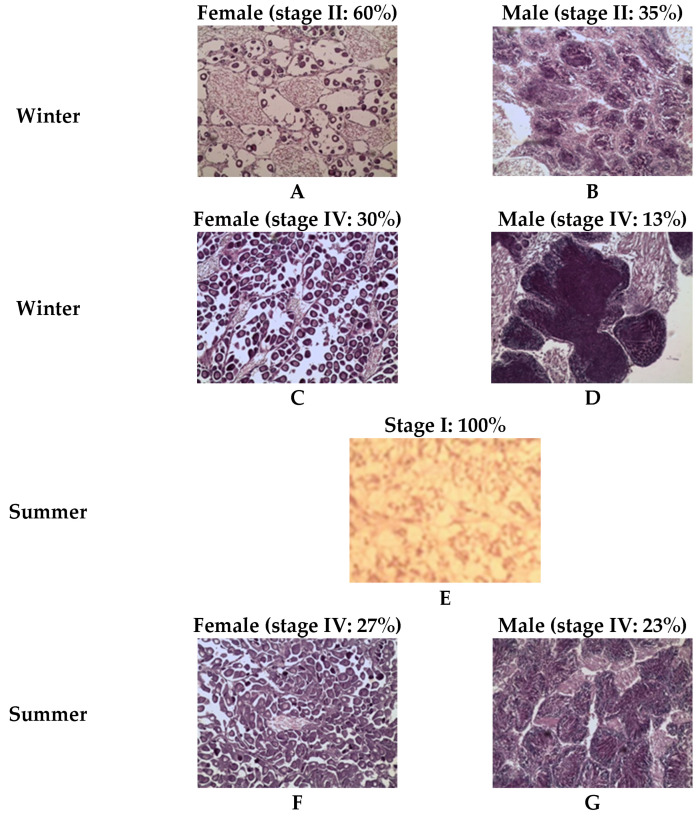
Histological examination showing the development of the grooved carpet shell clam *Ruditapes decussatus* gonad during winter and summer conditioning of the Atlantic population. (**A**) female during winter (week 0); (**B**) male during winter (week 0); (**C**) female during winter (week 9); (**D**) male during winter (week 9); (**E**) summer (week 0); (**F**) female summer (week 4); and (**G**) male during summer (week 4).

For the southern population (Atlantic Ocean), winter samples at week zero (W0) showed stage II of gonadal development, with a high proportion of the females (60%) and only 35% of the males. By week nine (W9), the southern clams (Atlantic Ocean population) had gonads in stage IV (ripe stage), with 30% of the females and 13% of the males in this stage (Figure 6). During summer, the Atlantic population (southern one) exhibited a homogeneous stage I across 100% of the individuals at week zero (W0), marked by a light color and simple structure (Figure 6). In summer, by the end of the experiment (week four (W4)), both female and male gonads of the Atlantic population (southern one) exhibited ripe stage characteristics, represented by 27% of the females and 23% of the males (Figure 6).

**Figure 7:**
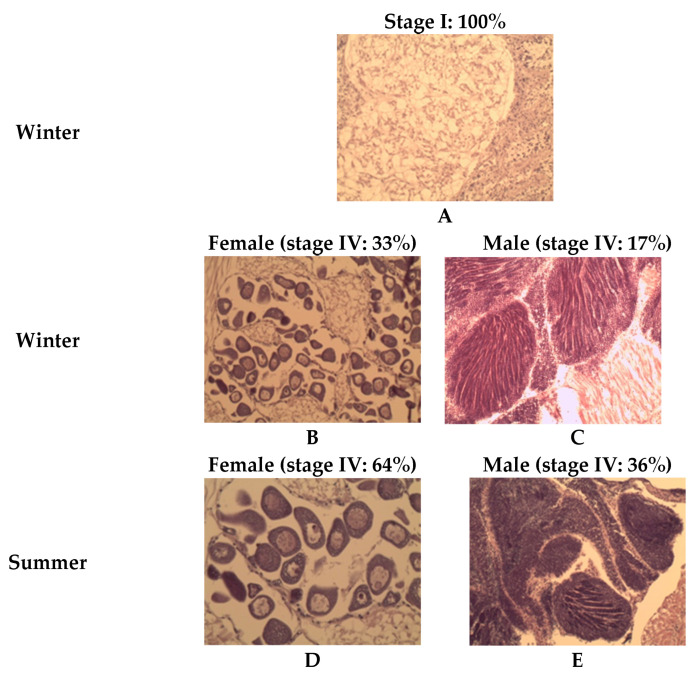
Histological examination showing the development of the grooved carpet shell clam *Ruditapes decussatus* gonad during winter and summer conditioning of the Mediterranean population. (**A**) winter (week 0); (**B**) female during winter (week 9); (**C**) male during winter (week 9); (**D**) female during summer (week 0); and (**E**) male during summer (week 0).

At the start of the winter conditioning (week zero) in the northern population (Mediterranean Sea), all samples were recorded at stage I (resting stage, (A)) (Figure 7). By the end of the winter conditioning period (week nine) in the northern population (Mediterranean Sea), 33% of the female samples reached stage IV (ripe stage), while only 17% of the male samples were at stage IV. However, during summer in the northern population (Mediterranean Sea), high percentages of female and male gonads in stage IV were observed compared to the winter season, reaching 64% and 36% for the females and males, respectively.

### 3.4. Spawning Induction

The highest percentage of spawners was obtained after nine weeks of conditioning during the winter period (69% for the Atlantic population), whereas only 42% of the Mediterranean ones responded to the spawning induction (Table 1). During the summer period, the successful spawning induction was quite similar for both populations (42% and 40% for Mediterranean and Atlantic clams, respectively).

In terms of the female spawners, the highest percentage was achieved for the Atlantic population (56%) during the winter conditioning (Table 1). In contrast, a low percentage of female spawners was obtained during the summer conditioning for the Atlantic population and during the summer and winter conditioning for the Mediterranean population (19%, 18%, and 17%, respectively). A high variability was observed in the number of oocytes released per female, ranging from 0.11 (×10^6^) in the Mediterranean clams during summer to 2.34 (×10^6^) during the winter conditioning in the same population. For the Atlantic population, the number of eggs released per female was higher during the winter conditioning with a value of 1.68 (×10^6^) compared to summer with 0.84 (×10^6^). The results of a two-way ANOVA showed that the number of oocytes released per female was significantly influenced by the origin (F = 16.35, *p* = 0.00028) and the season of collection (F = 19.75, *p* = 0.00033).

## 4. Discussion

### 4.1. Maturity Development

The gonadal development and the condition index are considered as the key indicators to evaluate the sexual maturation [17,26,47,48,49,50]. The conditioning and maturation of broodstock are crucial steps for the proper functioning of shellfish hatcheries. For clams, the maturation is controlled by several fundamental factors, such as the nutrition [23], temperature, and photoperiod [1,20,34,35,36,37,38], which mainly affect the gonad development. In this study, we investigated the effect of the season of broodstock collection (winter and summer) and the origin of clams collected from two different (oceanographically) sites of Morocco (North, Mediterranean Sea and South, Atlantic Ocean) on the broodstock performance in a hatchery. The results showed that, under controlled conditions, the time necessary to complete gametogenesis is interrelated with the collecting season as well as the broodstock origin. Based on the initial maturation indexes (CI and GCI) that were quite similar during the winter collection for clams from both localities, the maturation process in captivity was faster in the Mediterranean clams than in the Atlantic ones. The differences in gonadal development observed between the Mediterranean and Atlantic populations may be related to the different environmental conditions of each collection location (i.e., broodstock origin) [17], although potential genetic adaptations should also be considered, as has been previously described in the oyster *Crassostrea gigas* [51].

The differences in physiological indices observed between populations have been frequently associated with the influence of the geographical location and, consequently, by the inherent environmental factors such as the food availability, temperature, and salinity [34]. Despite a generally common reproductive cycle, some specific significant differences were observed between the gonadal developments in the two geographically distinct populations. Consequently, during summer, a short period of conditioning (four weeks) was required to improve the maturity for the Atlantic clams (from GCI = 2.90 ± 0.12 to GCI = 4.13 ± 1.21), while no conditioning was performed for the Mediterranean clams (successful spawning at week zero), due to the similarity of the gonadal state between the GCI = 4.13 ± 1.21 (week four) of the Atlantic population and the GCI = 4.31 ± 1.01 of the Mediterranean one at week zero. The effect of the collection period was evidenced while comparing gonadal condition index values at the end of each conditioning experiment. A significant statistical difference was found in the conditioning performance between the Mediterranean (northern) and Atlantic (southern) populations after 9 weeks during the winter conditioning period. The Mediterranean (northern) population achieved higher condition index (CI) and gonadal condition index (GCI) values of 13.60 and 6.01, respectively, compared to the Atlantic (southern) population, which recorded lower values of CI = 11.51 and GCI = 5.31. These results emphasize the superior physiological response of the Mediterranean population under identical conditioning conditions. The effect of broodstock origin on conditioning was previously demonstrated in *Ruditapes decussatus* from Portuguese wild beds [17].

### 4.2. Gametogenic Scale

Gametogenic cycles in bivalves are generally affected by the geographical location (i.e., origin of the broodstock) and, consequently, by the environmental factors. Temperature and food quantity and quality affect the gametogenic cycle of clams in wild beds. Different collection locations of the clams, when they are very distant, as the ones used in the present study are, may have very different environmental conditions and, therefore, the gametogenic cycle and periods of spawning of the clams may show differences [52]. We cannot exclude that an undetected spawning or earlier spawning took place in the wild before the collection of clams in the Atlantic population in summer, and thus it may explain the results of the gametogenic scales at week 0 for this population. Furthermore, Kamara et al. [27] showed that in Dakhla Bay there are two distinct spawning periods, from March to May and from July to September, which can explain the resting (stage I) of individuals during the summer samples (June) collected from this area. Several studies have indicated the existence of one single period of gamete release annually during summer for *Ruditapes decussatus* in Spain [53,54], Italy [55], and Ireland [56]. Other authors reported two major periods of spawning (spring and summer) in France [49,57] and Morocco [24]. Therefore, in light of these previous studies, local environmental factors seem to affect the gamete emission periods [58,59]. The southern Atlantic clams, at the start of the winter conditioning, were found to have 95% of the individuals in stage II (initiation of gametogenesis). However, after nine weeks of conditioning, 43% of the clams reached the ripe stage (stage IV) due to the adequate nutrition during the conditioning period, which provides the necessary energy and nutrients to support the gametogenic process, enabling the clams to progress to the reproductive stage effectively [48,60]. As well as this, a strict relationship between the condition index increments and the gonadal development was frequently observed by Matias et al. [17]. The reproductive dynamics of *Ruditapes decussatus* exhibit notable geographic variations, as highlighted by Kamara et al. [27], between the Atlantic (Dakhla) and Mediterranean (M’diq) populations. These differences are primarily driven by the distinct environmental conditions in each area. In Dakhla, located in the Atlantic, consistently warmer and more stable water temperatures result in two shorter spawning periods. Clams in Dakhla (Atlantic) complete gametogenesis and spawning in a short timeframe, leading to synchronized reproductive events, with 100% of the individuals observed in the resting stage during June, which is compatible with the results of our study. Conversely, in M’diq (Mediterranean), cooler and more variable water conditions extend the reproductive cycle. This slower progression of gametogenesis results in extended periods of maturity and staggered spawning events, likely as an adaptive response to the region’s fluctuating environmental conditions. During summer, clams in M’diq are in the ripe stage, reflecting this extended and gradual reproductive process. Our results in terms of the CI and GCI in summer at M’diq are in agreement with the results observed from the monitoring of the sexual maturity of *Ruditapes decussatus* in the M’diq natural bed carried out by the Shellfish station of Amsa (CI = 13.76 and GCI = 4.62).

At the beginning of the conditioning in the summer, the southern clams were in the sexual rest stage (stage I). Nevertheless, they reached maturity within just 4 weeks, likely due to conditioning factors like the temperature regime. Temperature plays a crucial role in regulating the gametogenic cycle in bivalves [46,61], and impacts their metabolism [20] by speeding up tissue metabolic processes, which in turn increases their need for food [60]. Furthermore, the gonadal maturation was also affected by food availability as well as the quality of the diet during the conditioning to achieve spawning in a short period [28,48].

The gametogenic state between the Atlantic (Dakhla) and Mediterranean (M’diq) areas exhibits notable differences, largely influenced by the distinct environmental conditions and geographic characteristics of each locality. The reproductive cycle of clams differs notably between the Atlantic and Mediterranean areas due to the contrasting environmental conditions in these regions. In the Atlantic region, the reproductive cycle is typically shorter, with clams completing gametogenesis and spawning within a more compressed time due to the consistently warmer water temperatures. This stable thermal environment promotes earlier and more synchronized reproductive events, which can explain the observed gametogenic state during the summer season, with 100% of the individuals recorded at the resting stage. On the other hand, in the Mediterranean area, the cooler and more fluctuating water temperatures extend the reproductive cycle, as gametogenesis progresses more slowly. The clams in this area may exhibit prolonged periods of maturity and staggered spawning, likely as an adaptation to the less predictable conditions, which can explain the ripe stage occurred during summer for this locality.

The Mediterranean population, at the beginning of the winter conditioning (W0), had 100% of the individuals in stage I (the resting stage). However, after nine weeks of broodstock conditioning, 50% of the clams achieved the ripe stage (stage IV) due to the positive energy balance from ingested food leading to the gonadal cells maturation [62]. During the summer, no conditioning was needed due to the successful spawning induction achieved at week zero (W0), which was attributed to their initial gonadal state (stage IV). This state was influenced by environmental factors in the wild, such as rising temperatures and chlorophyll-a levels, which positively affected the morphometric indexes [18].

For Moroccan hatcheries, the ability to produce clam spat independently of natural cycles could be highly valuable. This independence allows hatcheries to ensure continuous spat production, regardless of environmental or seasonal variations, and could be crucial for meeting market demands or addressing fluctuations in natural recruitment. Regarding oocyte quantities, while natural spawning may yield substantial quantities of oocytes during the peak of reproductive periods, conditioning may still provide a distinct advantage. By creating optimal conditions for gonadal development through controlled factors like the temperature, food supply, and photoperiod, conditioning can potentially enhance oocyte production beyond what is typically observed in natural conditions, while maintaining and improving gamete quality. Therefore, even if natural cycles may be sufficient under ideal circumstances, conditioning may still be necessary to consistently achieve high-quality and high-quantity oocyte production, especially when natural conditions are not favorable or when spat production needs to be standardized year-round.

### 4.3. Reproductive Output

Reproductive output is defined by three major components: the spawning success, number of released oocytes per female, and oocyte quality [61,62,63]. In our study, the spawning success varied between the four broodstock groups as a result of their differences in terms of collecting season and origin. A successful spawning induction was achieved after nine weeks of winter conditioning for the Mediterranean population, reaching 42% of the total response to spawning with 19% of the females releasing 2.34 million oocytes, which explains the efficiency of conditioning to increase the quality and number of oocytes released. During summer in the same Mediterranean population, 42% of the clams spawned (18% of the females spawned, releasing 0.11 million oocytes per female), without the need of conditioning due to the initial stage of gonads during this period (stage IV: ripe). For the Mediterranean population, despite the effective spawning induction without summer conditioning, only a low quantity of eggs per female was recorded (0.11 million). This could be explained by the low condition index (CI = 10.60 ± 1.02 and GCI = 4.31 ± 1.01) observed in the clams sampled at W0 in summer compared to the CI = 13.60 ± 1.02 and GCI = 6.01 ± 0.90 reached after the conditioning (W9) in winter. In the Atlantic population, after nine weeks of conditioning in winter, 69% of the clams spawned, and among them, 56% of the females responded positively, releasing 1.68 million eggs per female. This high spawning rate is likely attributed to the initial gonadal condition, with 90% of individuals already reaching stage II (gametogenesis initiation). At the beginning of the experiment (week 0) of the winter season, the gonadal condition index (GCI) in the natural beds was 2.77 for the Atlantic population and 3.00 for the Mediterranean ones, enabling a comparative analysis between the two populations. After the nine-week conditioning period, our findings showed a GCI of 5.31 and 6.01 for the Atlantic and Mediterranean populations, respectively. Previous GCI data monitored by Amsa Shellfish Station provided a reference point, indicating a GCI of 4.97 and 4.29 for the Atlantic and Mediterranean populations, respectively, in their natural environments. These results closely align with our findings and helped identify the approximate period for spawning induction when the clams reached maturity.

During the summer season, GCI values of week 0 were similar for both populations, with 4.11 for the Atlantic clams and 4.62 for the Mediterranean clams. Despite this similarity, the Mediterranean clams responded to the spawning induction immediately, while the Atlantic clams required four additional weeks of conditioning. Histological examination revealed that 100% of the Mediterranean clams were in the ripe stage, as noted in the results section. In contrast, the Atlantic clams were in the resting stage during this period, likely due to the post-spawning phase observed under natural environmental conditions, as reported by Kamara et al. [27]. However, the Mediterranean clams showed a low number of oocytes during this period (summer) and the Atlantic ones recorded a higher number of oocytes released, which indicates the importance of broodstock conditioning for improving the quantity of oocytes released during spawning operation.

In contrast, during summer, only 40% of spawners were obtained (17% of the female spawners releasing 0.84 million) after four weeks of conditioning, because of their initial gonadal stage (resting stage) which required the summer conditioning (4 weeks of broodstock conditioning) to achieve an earlier spawning induction.

An earlier spawning for clams is possible under certain conditions, but it essentially depends on environmental factors [63]. Under controlled hatchery environments, controlling the water temperature, photoperiod, and food availability can stimulate earlier gonad development and accelerate the reproductive process [20]. By providing a consistent, nutrient-rich diet and adjusting environmental cues, spawning could occur sooner than it would naturally. However, in wild populations, earlier spawning is due to fluctuating environmental conditions, and prematurely warm water or climate change might lead to mismatched timing with food availability [64]. This suggests that the combination of geographical origin and season was particularly suitable for supporting gamete production [17].

## 5. Conclusions

The current research enhanced our understanding of Moroccan clam production, focusing specifically on key aspects of gametogenesis. Our experiments, investigating the impact of the geographical origin and timing of collection on broodstock performance of *Ruditapes decussatus*, showed that there were significant differences in gametogenesis as well as in the spawning output between the two most important populations in Morocco. Both populations are of interest as broodstock sources for intensive clam hatchery production, due to the extended spawning period observed in both populations. The results of our study suggest that localities and the season of broodstock collection have a direct impact on the maturity development, through the initial ‘gonadic state’. Moreover, the period of conditioning plays a crucial role on maturity development due to the positive correlation with gonadal development, regarding the spawning rate and number of oocytes recorded.

The findings of this study provide valuable insights that can significantly enhance the production of clams, since this activity is very emergent and becoming very prominent in Morocco. By identifying key differences in the reproductive cycles and spawning patterns between distinct regions and seasons, the study offers critical data for optimizing broodstock management practices. Therefore, these findings can be largely considered as a baseline to plan *Ruditapes decussatus* production cycles in hatcheries. Additionally, understanding the influence of environmental factors from different geographic origins on reproduction may enable producers to adapt farming strategies to regional conditions, improving their overall productivity.

Further research on the broodstock conditioning of clams in Moroccan hatchery production could focus on optimizing environmental and nutritional parameters to enhance the reproductive performance. Studies could investigate the effects of varying the diet composition, including microalgae species and nutrient enrichment, on gametogenesis and spawning success. Additionally, research could explore the impact of precise temperature and photoperiod manipulations to better simulate natural cycles and improve conditioning efficiency. Genetic studies may also provide insights into population specific adaptations, enabling selective breeding programs to develop robust broodstock suited to local conditions. Moreover, the long-term monitoring of environmental factors, such as climate variability, could help refine conditioning protocols to mitigate potential disruptions. Such research would contribute to improving the sustainability and productivity of clam hatchery operations in Morocco.

## Figures and Tables

**Table 1:** Spawning characteristics of the grooved carpet shell clam *Ruditapes decussatus* under the different experimental origins and seasons.

Population	Spawning Parameters	Experiment (Season of Broodstock Collection)	
		Winter	Summer
South Atlantic	Spawners (%)	69	40
	Female spawners (%) Oocytes per female	56 1.68 ± 0.23 (10^6^)	17 0.84 ± 0.11 (10^6^)
North Mediterranean	Spawners (%)	42	42
	Female spawners (%) Oocytes per female	19 2.34 ± 0.28 (10^6^)	18 0.11 ± 0.10 (10^6^)

## Data Availability

All data generated are contained either in the article or extendable upon request to the corresponding author.
